# Hemorrhagic disseminated intravascular coagulation after ^177^Lu-Dotatate in metastatic midgut neuroendocrine tumor: A case report

**DOI:** 10.1097/MD.0000000000027455

**Published:** 2021-10-08

**Authors:** Noémie Worbe, Louise Damian, Véronique Le Cam-Duchez, Hervé Levesque, Pierre Michel, Elske Quak

**Affiliations:** a Rouen University Hospital, Department of Internal Medicine, Rouen, France; b Normandie Univ, UNIROUEN, Inserm U1096, Rouen University Hospital, Hemostasis Unit, Rouen, France; c Rouen University Hospital, Department of Hepato-gastroenterology, Rouen, France; d UNICANCER, Comprehensive Cancer Centre F. Baclesse, Department of Nuclear Medicine, Caen, France.

**Keywords:** ^177^Lu-Dotatate, case report, disseminated intravascular coagulation, neuroendocrine tumor, peptide receptor radionuclide therapy

## Abstract

**Rationale:**

Peptide receptor radionuclide therapy with ^177^Lu-Dotatate represents a major breakthrough in the treatment of metastatic well differentiated neuroendocrine tumors. This treatment is generally well tolerated. Reported severe long-term hematological side effects are rare and include hematopoietic neoplasms and bone marrow failure.

**Patients concerns:**

We describe the case of a patient presenting spontaneous bleeding and bruising occurring 6 weeks after the first administration of ^177^Lu-Dotatate. Blood tests showed anemia, thrombocytopenia, prolonged clotting times, profound fibrinolysis and low levels of coagulation factors II and V. There were no signs of tumor lysis syndrome.

**Diagnoses:**

We made the diagnosis of acute disseminated intravascular coagulation.

**Intervention:**

Treatment consisted of multiple transfusions of fresh frozen plasma, fibrinogen and platelets, and corticosteroids. Acute disseminated intravascular coagulation (DIC) persisted for 10 days and then resolved.

**Outcomes:**

Metabolic imaging 5 months after the ^177^Lu-Dotatate administration showed disease progression. Treatment with 177Lu-Dotatate was not rechallenged due to the occurrence of DIC.

**Lessons:**

Our case suggests that acute hemorrhagic disseminated intravascular coagulation can be a rare and life-threatening subacute side effect of ^177^Lu-Dotatate peptide receptor radionuclide therapy.

## 1 Introduction

Targeted treatment with the radiolabeled somatostatin analogue ^177^Lu-Dotatate represents a breakthrough in the systemic treatment of metastatic well differentiated neuroendocrine tumors (WD-NET), as most classic anticancer treatments have minor efficacy in this disease. In the NETTER-1 trial, progression free survival, overall response rate and quality of life were markedly improved in midgut NET patients treated with ^177^Lu-Dotatate as compared to a high dose of somatostatin analogues, validating ^177^Lu-Dotatate as second-line systemic therapy.^[1]^ In the main clinical trials, subacute grade 3 to 4 hematological toxicity is reported in 11% of patients.^[1,2]^ Long-term hematological toxicity (hematopoietic neoplasms or bone marrow failure) occurs in 1% to 4% of cases after a latency period of several years.^[3,4]^ No acute disseminated intravascular coagulation (DIC) has been reported.

## 2 Case report

Written informed consent was obtained from the patient for publication of this case report.

We describe the case of a fifty five-year-old woman who developed spontaneous bleeding and bruising 6 weeks after the first ^177^Lu-Dotatate administration. She had been diagnosed 10 years earlier with metastatic ileal WD-NET. She had no other medical history except controlled hypertension. Previous treatment lines included surgery, somatostatine analogues and transarterial chemoembolization of hepatic metastases. As the disease progressed despite previous treatment lines and ongoing therapy with somatostatine analogues, a treatment with ^177^Lu-Dotatate was initiated thirteen months after the last transarterial chemoembolization. Pretreatment blood tests were normal. She received a standard dosage of 7.4 Gigabecquerel intravenously. The first injection was administered without immediate side effects. Blood tests at week 2 and 4 after treatment showed grade 1 thrombocytopenia. At week 6, she presented to the emergency department with spontaneous gingivorrhagia and bruising. She had no fever; her general condition was good and clinical examination was unremarkable except for gum bleeding and multiple cutaneous ecchymosis. Initial laboratory tests showed normocytic regenerative anemia (hemoglobin 85 g/dL), thrombocytopenia (54 × 10.9 /L), no signs of hemolysis, no schistocytes, prolonged prothrombin time (25.8 seconds, 35%) and prolonged activated partial thromboplastin time (1.63), low fibrinogen (0.3 g/L), elevated fibrin monomer (>400 μg/mL) and elevated fibrin degradation products (>20 μg/mL). Coagulation factors II (49%) and V (44%) were low; factors VII (97%) and X (81%) were normal. Liver function tests were normal with the exception of moderate elevation of gamma Glutamyl Transferase (γGT = 100 UI/L, N < 38 UI/L). C-reactive protein was <5 mg/L. There was no sign of tumor lysis syndrome. The bone marrow aspirate and medullar karyotype were normal. Computed tomography showed stable metastatic disease, including a large stable necrotic liver metastasis.

**Figure 1:**
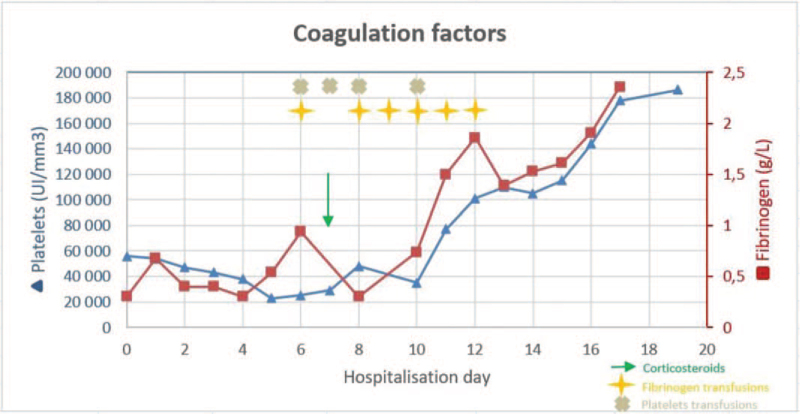
Evolution of platelet and fibrinogen levels.

Acute hemorrhagic DIC was diagnosed (International Society of Thrombosis and Haemostasis DIC Score = 8).^[5]^ For the next 10 days, as her clinical situation worsened with bleeding manifestations, including a voluminous compressive chest wall hematoma following bone marrow aspiration despite preventive fibrinogen transfusion, 10 fresh frozen plasma, 6 fibrinogen and 4 platelet transfusions were administered (Fig. 1). Because of the ineffectiveness of fibrinogen concentrates, we investigated and eliminated the presence of an anti-fibrinogen antibody.

Prednisone 40 mg/day was started on day 7 to reduce possible DIC-aggravating cancer or radiation related inflammation.

The evolution was favorable under corticosteroids and fibrinogen replacement therapy leading to normal hemostasis parameters and platelet count. Transfusions were stopped on day 11 and the patient was dismissed on day 13.

Treatment with ^177^Lu-Dotatate was not restarted. Metabolic imaging 5 months after the ^177^Lu-Dotatate administration showed disease progression.

## 3 Discussion

To our knowledge, this is the first report of acute DIC as a life-threatening subacute side effect of ^177^Lu-Dotatate peptide receptor radionuclide therapy in a patient with metastatic midgut WD-NET. The acute hemorrhagic DIC due to consumption coagulopathy required hemostatic treatment by multiple transfusions and fibrinogen injections. Recovery was slow. Causal link to the patient's long-term concomitant anti-hypertensive treatment (lercanidipine, irbesartan, and hydrochlorothiazide) was ruled out.

Several mechanisms might explain the occurrence of acute DIC in our patient. Firstly, cancer itself can activate the coagulation pathways and patients with cancer are at increased risk of thrombotic complications. However, DIC associated with solid tumors is usually chronic and indolent. Chronic DIC is typically treated with therapeutic doses of heparin.^[6,7]^ For our patient, there were no pretherapeutic signs of indolent DIC that might have aggravated due to treatment. In the literature, we found 2 cases of disseminated intravascular coagulation in metastatic neuroendocrine cancer, both associated with a pancreatic primary tumor. In 1 case, the patient died of DIC due to tumor lysis syndrome after disease manipulation by core biopsy and cytotoxic therapy. In the other case, DIC was reported to be related to the metastatic disease, and not a side effect of antineoplastic treatment. The DIC resolved after cytotoxic treatment.^[8]^

Aggressive hemorrhagic DIC can be associated with hematological malignancies at an early stage, such as acute promyelocytic leukemia, and is characterized by marked hyperfibrinolysis.^[9]^ In this situation, treatment of the underlying cancer and hematologic support can reverse the coagulopathy. Prothrombin time and platelets must always be monitored, as well as fibrinogen and fibrin degradation products. In case of bleeding, guidelines recommend the transfusion of platelet concentrates and plasma. Antifibrinolytic agents could also be effective in this situation. Fibrinogen concentrate substitution therapy is not recommended as first-line treatment of this type of DIC.

Secondly, for our patient, as the onset of DIC was rapid and severe and the metastatic midgut neuroendocrine tumor was stable, we supposed ^177^Lu-Dotatate was responsible for the consumption coagulopathy. DIC induced by antineoplastic treatment in solid cancers has been described, for example with vemurafenib in metastatic melanoma.^[10]^ We also hypothesized tumor necrosis induced by ^177^Lu-Dotatate could have stimulated the coagulation cascade. Tumor lysis syndrome is a rare complication of cancer therapy leading to metabolic abnormalities including hyperkalemia, hypophosphatemia, hyperuricemia and hypocalcemia frequently associated with severe kidney injury. Pro-inflammatory cytokines could be the link between tumor lysis syndrome and DIC. However, our patient had no biological and imaging signs of tumor lysis syndrome induced by ^177^Lu-Dotatate.

Lastly, the question remains whether the administration of the beta and gamma emitting radioisotope ^177^Lutetium could have provoked radiation induced DIC. Radiation induced DIC is described in animal experiments as part of space radiation research.^[11]^ In humans, bleeding and clotting disorders have been studied and described in atomic bomb casualties and victims of accidental whole-body irradiation.^[12]^ Acute radiation induced DIC due to the internal use of radioisotopes in medicine, some of which have been in use for decades, has only been reported in 2 cases of ^89^Strontium treatment for prostate cancer bone metastases.^[13,14]^ Therefore, as radiation can induce activation of the coagulation cascade resulting in DIC, the hypothesis of radiation induced DIC cannot be refuted. Moreover, as radiation is known to provoke inflammation, and inflammation in its turn can activate the coagulation cascade, this might explain why our patient responded so well to corticosteroid treatment.

Although in this single patient case no definite conclusions can be drawn concerning the cause of acute DIC, which was probably multifactorial, alertness is warranted. As the field of peptide receptor radionuclide therapy is expanding and other targeted radionuclide therapies are on their way in less-rare cancer types, as for example ^177^Lu- prostate-specific-membrane-antigen targeting prostate-specific-membrane-antigen in metastatic prostate cancer, the frequency of this rare and possibly fatal subacute side effect might increase in the coming years.

## Acknowledgments

The authors thank Alisson Johnson for her assistance in drafting the manuscript.

## Author contributions

**Conceptualization:** Louise Damian.

**Supervision:** Véronique Le Cam-Duchez, Hervé Levesque, Pierre Michel, Elske Quak.

**Validation:** Véronique Le Cam-Duchez, Hervé Levesque, Pierre Michel, Elske Quak.

**Writing – original draft:** Noémie Worbe, Louise Damian, Elske Quak.

**Writing – review & editing:** Louise Damian, Elske Quak.

## References

[1] Strosberg J El-Haddad G Wolin E. Phase 3 Trial of 177Lu-Dotatate for Midgut Neuroendocrine Tumors. N Engl J Med 2017;376:125–35. Doi: 10.1056/NEJMoa1607427. 28076709 10.1056/NEJMoa1607427 PMC5895095

[2] Bergsma H Konijnenberg MW Kam BLR. Subacute haematotoxicity after PRRT with 177Lu-DOTA-octreotate: prognostic factors, incidence and course. Eur J Nucl Med Mol Imaging 2016;43:453–63. 26419852 10.1007/s00259-015-3193-4 PMC4731438

[3] Bergsma H Lom Kvan Konijnenberg M. Therapy-related hematological malignancies after peptide receptor radionuclide therapy with 177Lu-DOTA-Octreotate: incidence, course & predicting factors in patients with GEP-NETs. J Nucl Med 2017;Published online August 3. jnumed.117.189712. doi:10.2967/jnumed.117.189712. 10.2967/jnumed.117.189712 28775205

[4] Brabander T Zwan WAvan der Teunissen JJM. Long-term efficacy, survival, and safety of [177Lu-DOTA0, Tyr3] octreotate in patients with Gastroenteropancreatic and Bronchial neuroendocrine tumors. Clin Cancer Res 2017;23:4617–24. 28428192 10.1158/1078-0432.CCR-16-2743

[5] Bakhtiari K Meijers JCM de Jonge E Levi M. Prospective validation of the International society of Thrombosis and Haemostasis scoring system for disseminated intravascular coagulation. Crit Care Med 2004;32:2416–21. 15599145 10.1097/01.ccm.0000147769.07699.e3

[6] Wada H Matsumoto T Yamashita Y. Diagnosis and treatment of disseminated intravascular coagulation (DIC) according to four DIC guidelines. J Intensive Care 2014;2:15 doi: 10.1186/2052-0492-2-15. 25520831 10.1186/2052-0492-2-15 PMC4267589

[7] Levi M. Management of cancer-associated disseminated intravascular coagulation. Thromb Res 2016;140:S66–70. 27067981 10.1016/S0049-3848(16)30101-3

[8] Davies RS Wells T Gwynne S. Disseminated intravascular coagulation in a patient with metastatic pancreatic neuroendocrine tumor: a case report and review of the literature. Case Rep Clin Med 2014;3:549–53.

[9] Levi M. Clinical characteristics of disseminated intravascular coagulation in patients with solid and hematological cancers. Thromb Res 2018;164:S77–81. 29703488 10.1016/j.thromres.2018.01.016

[10] van den Brom RRH Mäkelburg ABU Schröder CP de Vries EGE Hospers GAP. Vemurafenib-induced disseminated intravascular coagulation in metastatic melanoma. J Clin Oncol 2014;33:e133–4. 24778390 10.1200/JCO.2013.51.4471

[11] Krigsfeld GS Savage AR Billings PC Lin L Kennedy AR. Evidence for radiation-induced disseminated intravascular coagulation as a major cause of radiation-induced death in ferrets. Int J Radiat Oncol Biol Phys 2014;88:940–6. 24495588 10.1016/j.ijrobp.2013.12.001 PMC4039181

[12] Kennedy AR Maity A Sanzari JK. A review of radiation-induced coagulopathy and new findings to support potential prevention strategies and treatments. Radiat Res 2016;186:121–40. 27459701 10.1667/RR14406.1 PMC5041298

[13] Leong C McKenzie MR Coupland DB Gascoyne RD. Disseminated intravascular coagulation in a patient with metastatic prostate cancer: fatal outcome following strontium-89 therapy. J Nucl Med Off Publ Soc Nucl Med 1994;35:1662–4. 7931669

[14] Paszkowski AL Hewitt DJ Taylor AJ. Disseminated intravascular coagulation in a patient treated with strontium-89 for metastatic carcinoma of the prostate. Clin Nucl Med 1999;24:852. 10551466 10.1097/00003072-199911000-00006

